# PTRF/Cavin-1 as a Novel RNA-Binding Protein Expedites the NF-κB/PD-L1 Axis by Stabilizing lncRNA NEAT1, Contributing to Tumorigenesis and Immune Evasion in Glioblastoma

**DOI:** 10.3389/fimmu.2021.802795

**Published:** 2022-01-06

**Authors:** Kaikai Yi, Xiaoteng Cui, Xing Liu, Yunfei Wang, Jixing Zhao, Shixue Yang, Can Xu, Eryan Yang, Menglin Xiao, Biao Hong, Chuan Fang, Chunsheng Kang, Yanli Tan, Qixue Wang

**Affiliations:** ^1^ Laboratory of Neuro-oncology, Tianjin Neurological Institute, Tianjin Medical University General Hospital, Key Laboratory of Post-Neuro Injury Neuro-repair and Regeneration in Central Nervous System, Ministry of Education, Tianjin, China; ^2^ Department of Neuro-Oncology and Neurosurgery, Tianjin Medical University Cancer Institute and Hospital, National Clinical Research Center for Cancer, Key Laboratory of Cancer Prevention and Therapy of Tianjin, Tianjin’s Clinical Research Center for Cancer, Tianjin, China; ^3^ Beijing Neurosurgical Institute, Department of Neurosurgery, Beijing Tiantan Hospital, Capital Medical University, Beijing, China; ^4^ Department of Neurosurgery, Affiliated Hospital of Hebei University, Baoding, China; ^5^ Key Laboratory of Precise Diagnosis and Treatment of Glioma in Hebei Province, Baoding, China; ^6^ Department of Neurosurgery, Tianjin Medical University General Hospital, Tianjin, China; ^7^ Department of Pathology, Affiliated Hospital of Hebei University, Baoding, China; ^8^ Department of Pathology, Hebei University School of Basic Medical Sciences, Baoding, China

**Keywords:** glioblastoma, PTRF, lncRNA *NEAT1*, PDL1, immunosuppression

## Abstract

**Background:**

Immunotherapy, especially checkpoint inhibitors targeting PD-1 or PD-L1, has revolutionized cancer therapy. However, PD-1/PD-L1 inhibitors have not been investigated thoroughly in glioblastoma (GBM). Studies have shown that polymerase 1 and transcript release factor (PTRF/Cavin-1) has an immune-suppressive function in GBM. Thus, the relationship between PTRF and PD-L1 and their role in immune suppression requires further investigation in GBM.

**Methods:**

We used public databases and bioinformatics analysis to investigate the relationship between PTRF and PD-L1. We next confirmed the predicted relationship between PTRF and PD-L1 in primary GBM cell lines by using different experimental approaches. RIP-Seq, RIP, ChIP, and qRT-PCR were conducted to explore the molecular mechanism of PTRF in immunosuppression.

**Results:**

We found that PTRF stabilizes lncRNA *NEAT1* to induce NF-κB and PD-L1 and promotes immune evasion in GBM. PTRF was found to correlate with immunosuppression in the public GBM databases. PTRF increased the level of PD-L1 in primary cell lines from GBM patients. We carried out RIP-Seq of GBM cells and found that PTRF interacts with lncRNA *NEAT1* and stabilizes its mRNA. PTRF also promoted the activity of NF-κB by suppressing UBXN1 expression *via NEAT1* and enhanced the transcription of PD-L1 through NF-κB activation. Finally, PTRF promoted immune evasion in GBM cells by regulating PD-1 binding and PD-L1 mediated T cell cytotoxicity.

**Conclusions:**

In summary, our study identified the PTRF-*NEAT1*-PD-L1 axis as a novel immune therapeutic target in GBM.

## Introduction

Glioblastoma (GBM) is the most aggressive primary brain tumor in adults (1). Standard treatment regimens, including surgery, temozolomide chemotherapy, and radiation therapy, fail to improve patient survival (2). Immunotherapy, particularly immune checkpoint blockade with anti-programmed death 1 (PD-1)/PD-1 ligand (PD-L1) antibodies, has radically changed the treatment of multiple tumors (3–5). However, clinical studies investigating the use of anti-PD-1/PD-L1 in GBM remain are limited (6).

PD-L1 is an important immune checkpoint molecule on cancer cells which plays a critical role in immune surveillance and immune evasion. The majority of GBM patients are resistant to anti-PD-1/PD-L1 therapy, and only a minority of patients respond to this immunotherapy (7). The response to PD-1/PD-L1 blockade is correlated with PD-L1 expression in tumor cells (8, 9). Previous studies demonstrated that PD-L1 protein levels are associated with glioma grades, with elevated levels on tumor cells facilitating immune evasion in glioma patients (10, 11). Thus, one possible mechanism of immune evasion in GBM is the upregulation of PD-L1 which requires an in-depth investigation. Recent studies show that the expression of PD-L1 in tumors is regulated by signaling pathways, transcription factors and epigenetic factors, such as the Wnt/β-catenin pathway (12), STAT3 (13), and NF-κB (14). To develop an effective strategy to enhance antitumor immunity, the underlying mechanisms of PD-L1 regulation in GBM need to be elucidated.

Polymerase 1 and Transcript Release Factor (PTRF, also known as Cavin-1) is upregulated in GBM with mesenchymal phenotype compared with other subtypes of GBM (15). The prognosis of GBM patients with high PTRF expression is also worse (15). Previous studies found that PTRF protects vascular endothelial cells from cytotoxic T cell-mediated lysis (16). Further, in the challenge phase of asthma, loss of PTRF led to intense airway inflammation and potent type 2 immune responses (17). In GBM, bioinformatics analysis showed a negative correlation between PTRF expression and cytotoxic lymphocytes (18). We also demonstrated that PTRF overexpression increased tumor proliferation and decreased tumor-infiltrating CD8^+^ T cells mediated anti-tumor immunity (19). However, the mechanism by which PTRF influences immunosuppression, particularly PD-L1 expression, in GBM needs further research.

Here, we show that PTRF correlates with immunosuppression and increases the expression of PD-L1 in GBM. Further we found that PTRF interacts with lncRNA *NEAT1* and maintains the mRNA stability of *NEAT1*, increasing H3K27me3 level on the UBXN1 promoter, which results in the binding of NF-κB to the PD-L1 promoter. These findings highlight the role of the PTRF-*NEAT1*-PD-L1 axis in immune evasion in GBM; this immune-suppressive axis may be targeted as a novel immunotherapeutic strategy.

## Materials and Methods

### Cell Culture, Lentiviruses, and Chemicals

N9 cell lines derived from GBM patients, were established by Professor Fan of Beijing Normal University. TBD0220 cell lines derived from GBM patients, were established by Professor Kang of Tianjin Medical University (20, 21). N9 and TBD0220 cells were cultured in DMEM/F12 supplemented with 1% Penicillin-Streptomycin and 10% FBS. Cells were maintained at 37°C in a humidified 5% CO2 atmosphere. All GBM cells, except *in vivo* cultures, were maintained for less than eight generations. Lentiviruses encoding the PTRF-EGFP fusion protein (GOSL87854) and luciferase (GCNL84615) were purchased from GENECHEM (Shanghai, China). Small interfering RNAs targeting PTRF were synthesized by GenePharma (Shanghai, China). Sequences of siRNAs were as follows: PTRF siRNA#1 5’-GCCGCAACUUUAAAGUCAUGAUCUA; PTRF siRNA#2 5’-AGGAGUCCCGCGCAGAGCGUAUCAA. Small interfering RNAs targeting *NEAT1* were synthesized by GenePharma (Shanghai, China). Sequences of siRNAs were as follows: *NEAT1* siRNA 5’- GAACUUUACUUCGUUAGAUTT. We treated cells with Actinomycin D (Selleck, S7418) at a final concentration of 1 μM.

### Western Blot Analysis

The protein was extracted GBM cells by RIPA protein lysis buffer (Solarbio, R0010), with a freshly added mixture of protease inhibitor cocktail and PMSF. Then, 20 µg of protein was run on an SDS-PAGE gel, separated by electrophoresis and transferred to PVDF membranes. The membrane was first blocked by 5% BSA for 1-2 hours at room temperature, followed by incubation with primary antibodies at 4°C overnight. After washing three times with PBST, the membrane was blocked with secondary antibodies (Promega, 1:10000). Protein bands were detected was performed using G:BOXF3 (Syngene, UK) with Western HRP Substrate (Millipore, WBLUF0500). The antibodies used for western blots were: PD-L1 (Abcam, ab58810, 1:500), NF-κB (Cell Signaling Technology, 8242, 1:1000), Phospho-NF-κB (Cell Signaling Technology, 3033, 1:1000), UBXN1 (Proteintech, 16135-1-AP, 1:1000), GAPDH (Proteintech, 60004-1-Ig, 1:1000).

### Quantitative RT-PCR

Cells were lysed in TRIzol Reagent and mixed with chloroform, then centrifuged at 12000 rpm for 10 minutes at 4°C. The upper aqueous phase was transferred to a clean tube and an equal volume of isopropanol was added. The samples were mixed and stored at −20°C overnight. The concentration of total RNA was measured by NanoDrop 2000 (Thermo Scientific, USA). cDNA was synthesized from 1 µg of RNA using a reverse transcription kit (Promega, USA), according to the manufacturer’s protocol. qRT-PCR was performed using the DNA Engine Opticon 2 Two-Color qRT-PCR detection system (Bio-Rad Laboratories, USA) and the results were normalized to the GAPDH. The following primers were used:

*PD-L1*-F: AGGAGTACCTTGGCTTTGCC
*PD-L1*-R: GCCTTGCTCAGCCACAATTC
*NEAT1*-1F: CCAGTTTTCCGAGAACCAAA
*NEAT1*-1R: ATGCTGATCTGCTGCGTATG
*NEAT1*-2F: CTAGAGGCTCGCATTGTGTG
*NEAT1*-2R: GCCCACACGAAACCTTACAT
*UBXN1*-F: AGTGAAGAGGAAAGACAGGAA
*UBXN1*-R: TTTGTCCCTCTCGATCTTTTC
*TNFα*-F: CCCAGGCAGTCAGATCATCTTC
*TNFα*-R: GGTTTGCTACAACATGGGCTACA
*IL1β*-F: CCACAGACCTTCCAGGAGAATG
*IL1β*-R: GTGCAGTTCAGTGATCGTACAGG
*IL8*-F: GAGTGATTGAGAGTGGACCACACT
*IL8*-R: AGACAGAGCTCTCTTCCATCAGAAA
*GAPDH*-F: TGCACCACCAACTGCTTAGC
*GAPDH*-R: GGCATGGACTGTGGTCATGAG

### Flow Cytometry

For the detection of cell-surface PD-L1, cells were trypsinized and collected into a flow tube. The cells were incubated with 100 μL PBS by adding PE anti-human PD-L1 Antibody (329705, BioLegend; the dilution ratio is 1: 100) at room temperature for 1 hour. Then, the cells were analyzed *via* flow cytometer after washing with PBS.

### RNA-Binding Protein Immunoprecipitation

RIP experiment was performed using the Magna RIP RNA-Binding Protein Immunoprecipitation Kit (Millipore, No.17-701) according to the manufacturer’s instructions. For high-throughput sequencing (RIP-Seq), the libraries were prepared following the manufacturer’s instructions and applied to the Illumina HiSeq X Ten system by ABlife. Inc (Wuhan, China). To further validate the RIP-Seq results, the RNA fraction, precipitated by RIP, was analyzed *via* RT-qPCR. The data presented in the study are deposited in the SRA repository, accession number PRJNA777377

### Chromatin Immunoprecipitation

ChIP experiment was performed using the Magna ChIP Chromatin Immunoprecipitation Kit (Millipore, No.17-10085) according to the manufacturer’s instructions. The antibodies against H3K27me3 and NF-κB were purchased from Cell Signaling Technology (Denver, MA, USA). The primers used for UBXN1 have been described. The primers used for UBXN1 and PDL1 were as follows:

UBXN1-F: GGCAGGGGAAATGATGGATA
UBXN1-R: TAACCTGCCCACTCCATTAC
PD-L1-1F: ACTGAAAGCTTCCGCCGATT
PD-L1-1R: GAGGAACAACGCTCCCTACC
PD-L1-2F: GGGTGGCAGAATATCAGGGAC
PD-L1-2R: CGTGGATTCTGTGACTTCCTC
PDL1-3F: ACCTGTAAACTGTATTGCCACA
PDL1-3R: TGGTGACTGTAAGTTTGGGTGA
PDL1-4F: AGGGTAGAAACAGGTGGGAA
PDL1-4R: GAAAGCAGTGTTCAGGGTCTAC

### Luciferase Reporter Assay

The expression vectors encoding PGL3-Basic-PD-L1^WT^ and PGL3-Basic-PD-L1^MUT^ were synthesized by Integrated Biotech Solutions (Shanghai, China). Briefly, the GBM cells were transfected with 0.5 μg of PGL3-Basic empty vector, PGL3-Basic-PD-L1^WT^ and PGL3-Basic-PD-L1^MUT^ by Lipofectamine 3000 (Invitrogen) separately. After incubation for 48 hours, luciferase activity was measured using the luciferase assay system (E1501, Promega) according to the manufacturer’s protocol.

### *In Situ* Hybridization Histochemistry

The glioma tissues were surgically resected from patients at the Beijing Tiantan Hospital. The ISH experiments for *NEAT1* were performed using the RNA ISH kit (Boster, China) according to the manufacturer’s instructions. The probes were designed and synthesized by Boster (Wuhan, China).

### *In Vivo* Intracranial Patient-Derived Xenografts Model

Female BALB/C nude mice (4 weeks old) were used for intracranial GBM Patient-derived xenografts, and TBD0220 cells were used to construct the intracranial model. The cells were injected into the mice under the guidance of a stereotactic instrument at coordinates relative to bregma. Bioluminescence imaging was used to detect intracranial tumor growth by *In Vivo* Imaging System (IVIS) Spectrum. On day 21, the mice were sacrificed, and the brain tissues were removed. The brain tissue was then fixed in 10% formalin for 24 h, embedded in paraffin, and sectioned into 8 μm slices. All experiments were approved by the Animal Ethical and Welfare Committee of Tianjin Medical University. Kaplan-Meier survival curves were plotted to analyze the survival.

### HE and Immunohistochemical Staining

The glioma tissues were surgically resected from patients at the Beijing Tiantan Hospital. The tissues were embedded in paraffin, sectioned, and stained with HE. IHC staining was performed as described previously. Staining intensity was quantified by a pathologist. Antibodies include Ki-67 (ZSGB-BIO, ZM-0167, 1: 100), Phospho-NF-κB (Cell Signaling Technology, 3033, 1:400), UBXN1 (Proteintech, 16135-1-AP, 1:200), PTRF (Proteintech, 18892-1-AP, 1:200), PD-L1 (Cell Signaling Technology, 13684, 1:400), CD8 (Proteintech, 66868-1-lg, 1:200).

### PD-L1/PD-1–Binding Assay

Confocal laser scanning microscopy (CLSM) and flow cytometry were used to study the PD-L1/PD-1–binding in GBM cells. Cells were trypsinized and collected into a flow tube. Control and PTRF overexpressing TBD0220 cells were incubated with 5 μg/mL recombinant human PD-1 FC chimera protein (1086-PD-050, R&D Systems) at room temperature for 1 hour. After incubation, the cells were washed three times with PBS and incubated with anti-Human IgG (H+L) Cross-Adsorbed Secondary Antibody (Alexa Fluor 488) (A-11013, Thermo Fisher) for 1 hour at room temperature. For CLSM analysis, the cells were fixed with paraformaldehyde for 15 minutes at room temperature and further stained with DAPI for detecting the cell nucleus. CLSM images were observed by FV-1200 laser scanning confocal microscope (Olympus Corporation, Japan). For flow cytometry analysis, the cells were trypsinized and collected by centrifugation, then resuspended in PBS. The data were analyzed by FlowJo v10.6.2.

### T Cell Cytotoxicity Assay

Control and PTRF overexpressing TBD0220 cells were separately transfected with siRNA for *NEAT1* in DMEM/F12 medium. Human peripheral blood mononuclear cells (PBMC; CBP-002, StemEry) were maintained in DMEM/F12 medium. The PBMC cells were activated with 100 ng/mL CD3 antibody (317325, BioLegend), 100 ng/mL CD28 antibody (302933, BioLegend), and 10 ng/mL IL2 (589102, BioLegend) for 24 h. Then, activated PBMC cells were incubated with TBD0220 cells at 10:1 ratio in the presence of caspase-3/7 green detection reagent (C10723, Thermo Fisher, 1:1000) at 37°C for 12 hours according to the manufacturer’s protocol. The cell nuclei were visualized by staining with DAPI. All the images were observed using FV-1200 laser scanning confocal microscope (Olympus Corporation, Japan).

### Clinical Information And Transcriptome Data of Glioma Patients

The clinical and RNA sequencing data sets of glioma patients in TCGA database were downloaded from the University of California Santa Cruz (UCSC) Xena Functional Genomics Explorer (https://xenabrowser.net/). CGGA-693 RNA sequencing data sets and Rembrandt microarray data were obtained from the Chinese Glioma Genome Atlas (CGGA) (http://www.cgga.org.cn/). All the RNA sequencing data were transformed by log2 (normalized count + 1).

PTRF-correlated genes were obtained by a filtering condition: Pearson correlation coefficients between the expression of PTRF and other genes ≥ 0.3 and p-value ≤ 0.05. GO-BP analysis of PTRF-correlated genes was performed by the cluster Profiler v3.18.0 R package and visualized by bubble charts. Tumor purity of glioma data in TCGA, CGGA, and Rembrandt databases was measured by estimate v1.0.13 R package. The univariant and multivariant cox analyses were employed by survival v3.1.12 R package and displayed as forest maps. The PNP score of each patient was calculated by the formula below, and overall survival data of patients with PNP score-high or low were shown as survival curves.

β-value _(PTRF)_ × expression data _(PTRF)_ + β-value _(_ *_NEAT1_*_)_ × expression data _(_ *_NEAT1_*_)_ + β-value _(PD-L1)_ × expression data _(PD-L1)_

### Statistical Analysis

All data visualization was performed using Microsoft Excel, GraphPad Prism 8 software, and R. Two-tail unpaired Student’s t-test was used to compare two experimental groups, and one-way or two-way ANOVA was used when comparing three or more experimental groups. All data represent the mean ± SD. A p-value less than 0.05 was considered statistically significant, ns = not significant. Significance was defined as *p < 0.05, **p < 0.01, ***p < 0.001, ****p < 0.0001.

## Results

### PTRF Increases PD-L1 Levels and Correlates With Immunosuppression in GBM

**Figure 1:**
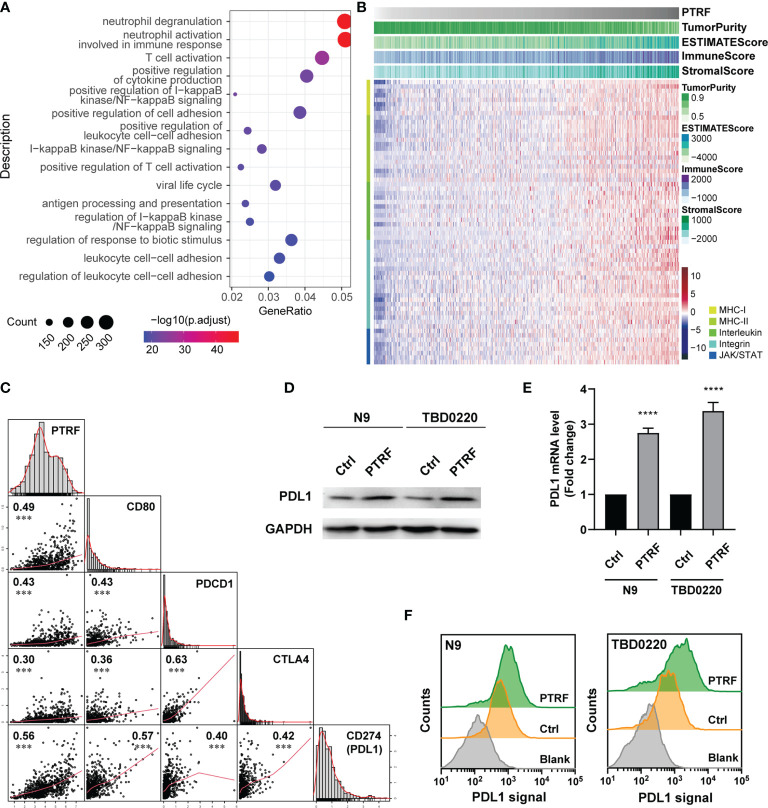
PTRF increases PD-L1 levels and correlates with immunosuppression in GBM. **(A)** Gene ontology enrichment analysis of genes that are positively associated with PTRF in the CGGA GBM database. **(B)** Heatmap showing the relationship between PTRF, low tumor purity, and genes regulating immunity in the CGGA GBM database. **(C)** The correlation between PTRF, CD80, PDCD1, CTLA4, and PD-L1 levels in the CGGA GBM database, measured by Pearson’s correlation test. **(D)** Western blot analysis of PD-L1 expression in primary GBM cells (N9 and TBD0220; both control and PTRF overexpression cells). **(E)** Relative mRNA level of PD-L1 in control and PTRF overexpressing cells. **(F)** Flow cytometry analysis of PD-L1 in control and PTRF overexpressing cells. ****p ≤ 0.001.

In this study, considering the immune-regulation function of PTRF, we assessed the relationships between PTRF and immune responses in GBM by examining GBM cases in public databases. We acquired the transcriptome data of glioma in CGGA, TCGA, and Rembrandt databases, and calculated the correlation between PTRF and other genes. The PTRF co-expression gene sets (r>0.3) were filtered out and interrogated by gene ontology (GO) enrichment analysis. GO-biological process (GO-BP) results show that the functions associated with T cell activation was significantly enriched (**Figure 1A** and **Supplemental Figure 1A**). Elevated expression of PTRF was associated with low tumor purity and had a positive correlation with the expressions of genes, involved in tumor immunity (**Figure 1B**, **Supplemental Figure 1B**). The correlation between PTRF and the representative genes of immune cells in TCGA, CGGA, and Rembrandt databases, were calculated and visualized as scatter maps (**Figure 1C** and **Supplemental Figure 1C**). We found that the expression of PTRF positively correlated with PD-L1 (correlation coefficient of 0.56), suggesting that PTRF may regulate PD-L1. To investigate the regulation of PD-L1 by PTRF, we stably overexpressed PTRF in primary GBM cell lines (N9 and TBD0220) by transfecting lentivirus. We conducted immunoblotting analysis of cultured N9 and TBD0220 cells and found that PTRF overexpression promoted the increase of PD-L1 protein levels in N9 PTRF and TBD0220 PTRF cells compared with their respective control cells (**Figure 1D** and **Supplemental Figure 1D**). Real-time quantitative PCR (RT-qPCR) determined that the mRNA level of PD-L1 was increased in N9 PTRF and TBD0220 PTRF cells compared with the control cells (**Figure 1E**).

We further employed flow cytometry to interrogate the PD-L1 level changes on the cell membranes of N9 and TBD0220 cells overexpressed PTRF. We confirmed that PD-L1 protein levels were higher in the PTRF overexpressing cells compared with the control cells (**Figure 1F**). We verified the effect of PTRF on PD-L1 by PTRF knock-down in N9 and TBD0220 cells. The mRNA and protein level of PD-L1 was significantly decreased by PTRF knockdown in N9 and TBD0220 cells compared with their respective control cells (**Supplemental Figures 1E, F**). These results show that PTRF regulates the expression of PD-L1 at the transcription level in GBM cells.

### PTRF Maintains mRNA Stability of lncRNA *NEAT1*

**Figure 2:**
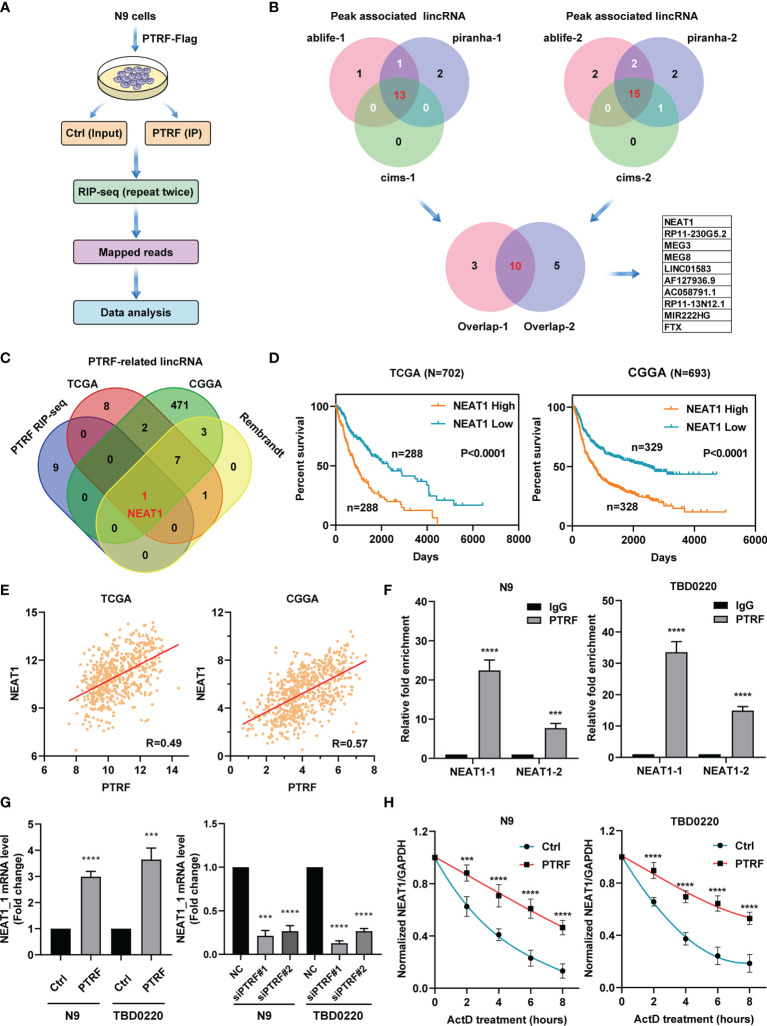
PTRF interacts with LncRNA *NEAT1* and maintains its mRNA stability. **(A)** Schematic of RIP-seq analysis in primary GBM cells with PTRF overexpression. **(B)** Venn diagrams showing the number of peaks associated with lncRNA that overlap between two repeated experiments by three computational methods: (a) CIMS, (b) Piranha, and (c) ABlife. Ten lncRNAs were identified as potential PTRF-associated RNA molecules. **(C)** Venn diagrams of results for potential lncRNAs between PTRF RIP-Seq, TCGA, CGGA, and Rembrandt database. **(D)** The Kaplan–Meier curves of patients with high or low lncRNA *NEAT1* expression (the TCGA database and CGGA database). **(E)** The correlation between PTRF and *NEAT1* levels in the TCGA and CGGA GBM database. **(F)** RIP-qPCR verification of RIP-Seq results with PTRF antibodies for the enrichment of *NEAT1* in N9 and TBD0220 cells. **(G)** The relative level of *NEAT1* in control and PTRF overexpressing cells (left). The level of *NEAT1* after PTRF knockdown in N9 and TBD0220 cells (right). **(H)** RT-qPCR analysis of *NEAT1*, treated with actinomycin D (ActD) at the indicated time points in N9 and TBD0220 cells. ***p ≤ 0.001 and ****p ≤ 0.001.

PTRF is a novel non-canonical RNA-binding protein (RBP) (22) and participates in the transcription regulation of PD-L1 in GBM. To investigate the potential mechanism of PTRF as an RBP affecting the expression of PD-L1, we performed RBP immunoprecipitation-coupled high-throughput sequencing (RIP-Seq) by Illumina HiSeq X Ten system in N9 cells with PTRF-Flag over-expression (**Figure 2A**). The experiments were biologically repeated twice and the clean reads were mapped to the human reference genome (GRCh38) using TopHat2. The reads were analyzed for PTRF-associated RNA sequences after normalization by three computational methods: (a) CIMS, (b) Piranha, and (c) ABlife. Based on screening with two replicates by three different computations, we identified ten long noncoding RNAs (lncRNAs) as potential PTRF-associated RNA molecules (**Figure 2B**).

To clarify the function of PTRF as RBP, we performed the co-expression analysis to identify PTRF-related lncRNAs (r > 0.3) in TCGA, CGGA, and Rembrandt glioma databases. We overlapped the lncRNAs data from RIP-Seq results and public GBM databases. The cross‐comparison of the four lists revealed that nuclear enriched abundant transcript 1 (*NEAT1*) is the lncRNA that interacts with PTRF (**Figure 2C**). Patients with a high level of *NEAT1* had shorter overall survival times compared with those with low *NEAT1* expression (**Figure 2D** and **Supplemental Figure 2A**). Further, we found a significant correlation between PTRF and *NEAT1* in TCGA (r = 0.49), CGGA (r = 0.57), and Rembrandt (r = 0.49) databases (**Figure 2E** and **Supplemental Figure 2B**). RBP immunoprecipitation–coupled RT-qPCR was performed in N9 and TBD0220 cells in which the cell lysates were followed by IP with the antibody against PTRF. Given that two transcripts of *NEAT1*, *NEAT1*_1 (3.7 kb) and *NEAT1*_2 (23 kb), we designed two pairs of primers for PCR amplification, respectively. Quantitative PCR analysis showed the enrichment of *NEAT1*_1 and *NEAT1*_2 in the PTRF group, compared with the IgG group, validating the RIP-Seq results. The enrichment of *NEAT1*_1 was significantly higher than that of *NEAT1*_2 in RBP immunoprecipitation (**Figure 2F**).

To verify the effect of PTRF on *NEAT1*, we conducted quantitative PCR analysis to determine that the mRNA level of *NEAT1*_1 was increased in N9 PTRF and TBD0220 PTRF cells than control cells. The mRNA level of *NEAT1*_1 was significantly decreased by PTRF knockdown in N9 and TBD0220 cells (**Figure 2G**). The effect of PTRF on *NEAT1*_2 is consistent with the results of *NEAT1*_1 (**Supplemental Figures 2C, D**). Considering the pivotal role of RBP in post-transcriptional regulation, we investigated whether PTRF regulated the stability of *NEAT1* with actinomycin D (ActD) blocking mRNA synthesis. We conducted a quantitative PCR–based time curve (0–8 h) analysis of *NEAT1*, which showed that PTRF resulted in significantly lower degradation of *NEAT1* level (**Figure 2H** and **Supplemental Figure 2E**).

### PTRF Suppresses UBXN1 Expression and Promotes the Activity of NF-κB *via NEAT1*

**Figure 3:**
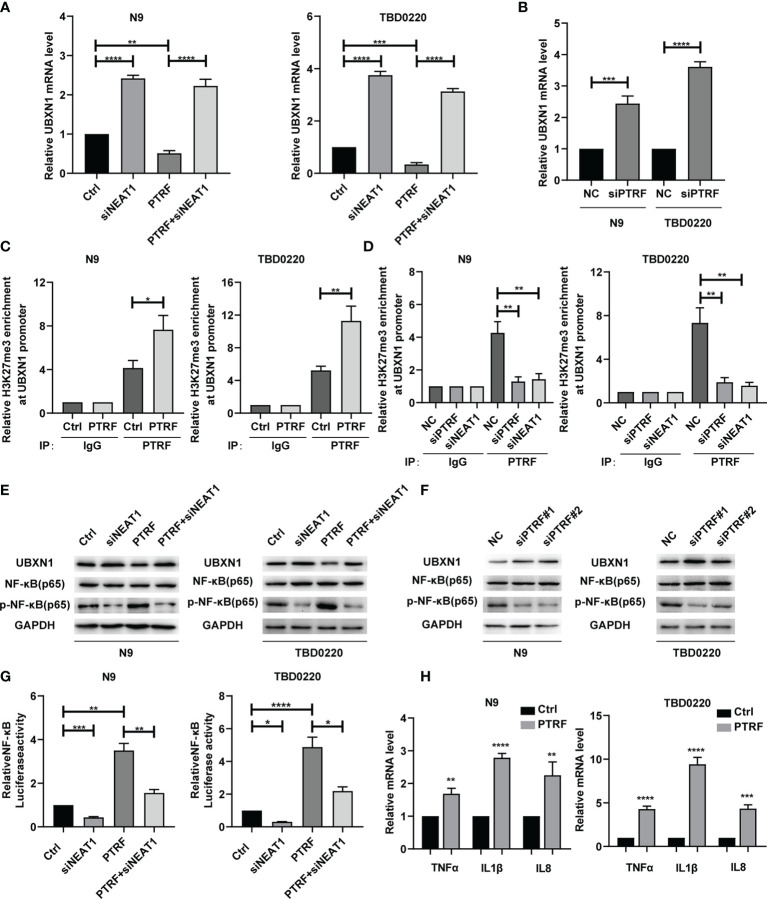
**(A)** RT-qPCR analysis of UBXN1 in N9 and TBD0220 cells with or without PTRF overexpression, as well as treatment with *NEAT1* knockdown. **(B)** The mRNA level of UBXN1 after PTRF knockdown in N9 and TBD0220 cells. **(C)** ChIP-PCR analysis of the ability of H3K27me3 to bind to the UBXN1 promoter using H3K27me3 antibody in N9 and TBD0220 cells with or without PTRF overexpression. **(D)** ChIP-PCR analysis of the ability of H3K27me3 to bind to the UBXN1 promoter normalized to IgG with PTRF or *NEAT1* knockdown in N9 and TBD0220 cells, respectively. **(E)** Western blot analysis of UBXN1, NF-κB, and P-NF-κB expression in primary GBM cells (N9 and TBD0220) with or without PTRF overexpression, as well as treatment with *NEAT1* knockdown respectively. **(F)** Western blot analysis of UBXN1, NF-κB, and P-NF-κB expression with PTRF knockdown in N9 and TBD0220 cells. **(G)** The Luciferase activity of NF-κB in N9 and TBD0220 cells with or without PTRF overexpression, as well as treatment with *NEAT1* knockdown respectively. **(H)** The mRNA level of NF-κB downstream target genes, including TNFα, IL-1β, and IL-8 in N9 and TBD0220 cells with PTRF overexpression. *p ≤ 0.05, **p ≤ 0.01, ***p ≤ 0.001, and ****p ≤ 0.001.

The genes that positively correlated with PTRF were found to be closely related to the NF-κB signaling pathway (**Figure 1A**). Since, NF-κB plays a crucial role in the immune response and PD-L1 regulation, we explored the role of *NEAT1* in the regulation of NF-κB in a PTRF-dependent manner. We previously demonstrated that the PRC2 complex promoted NF-κB activity *via* the trimethylation of H3K27 (H3K27me3) in the UBXN1 promoter (23, 24). Based on the interaction between *NEAT1* and EZH2, we designed siRNA to knock-down *NEAT1* expression. The level of UBXN1 was significantly decreased by PTRF overexpression in N9 and TBD0220 cells, and this decrease was reversed by *NEAT1* knockdown (**Figure 3A**). Further, the level of UBXN1 was significantly increased in PTRF knockdown cells compared with the control cells (**Figure 3B**). We next performed chromatin immunoprecipitation (ChIP)-qPCR analysis to evaluate the chromatin state of the UBXN1 promoter and found that the enrichment levels of H3K27me3 were increased at the UBXN1 promoter region in cells overexpressing PTRF (**Figure 3C**). Further, knockdown of PTRF or *NEAT1* reduced H3K27me3 enrichment at the UBXN1 promoter region in N9 and TBD0220 cells (**Figure 3D**).

We next investigated how PTRF regulates the activity of NF-κB. PTRF overexpression decreased the proteins level of UBXN1 (**Figure 3E** and **Supplemental Figure 3A**) and increased the nuclear levels of p-NF-κB in N9 and TBD0220 cells, which were reversed by knocking down *NEAT1* (**Figure 3E** and **Supplemental Figure 3B**). In contrast, knockdown of PTRF increased the UBXN1 proteins level (**Figure 3F** and **Supplemental Figure 3C**) and decreased the nuclear levels of p-NF-κB (**Figure 3F**). In addition, we cultured N9 and TBD0220 cells with lentivirus vector expressing a NF-κB luciferase construct. Luciferase reporter assay showed that the activity of NF-κB was significantly up-regulated by overexpression of PTRF in N9 and TBD0220 cells and the effect was reversed by *NEAT1* knockdown (**Figure 3G**). Knockdown of PTRF decreased the luciferase activity of NF-κB reporter (**Supplemental Figure 3D**). We also detected the expression of NF-κB target genes, confirming that NF-κB was activated by PTRF overexpression in N9 and TBD0220 cells (**Figure 3H**). PTRF knockdown produced the opposite effects (**Supplemental Figure 3E**). These results suggest that PTRF suppresses UBXN1 expression and promotes the activity of NF-κB *via NEAT1*.

### PTRF Contributes to GBM Proliferation Through the UBXN1/NF-κB Axis by Regulating *NEAT1 In Vivo*

**Figure 4:**
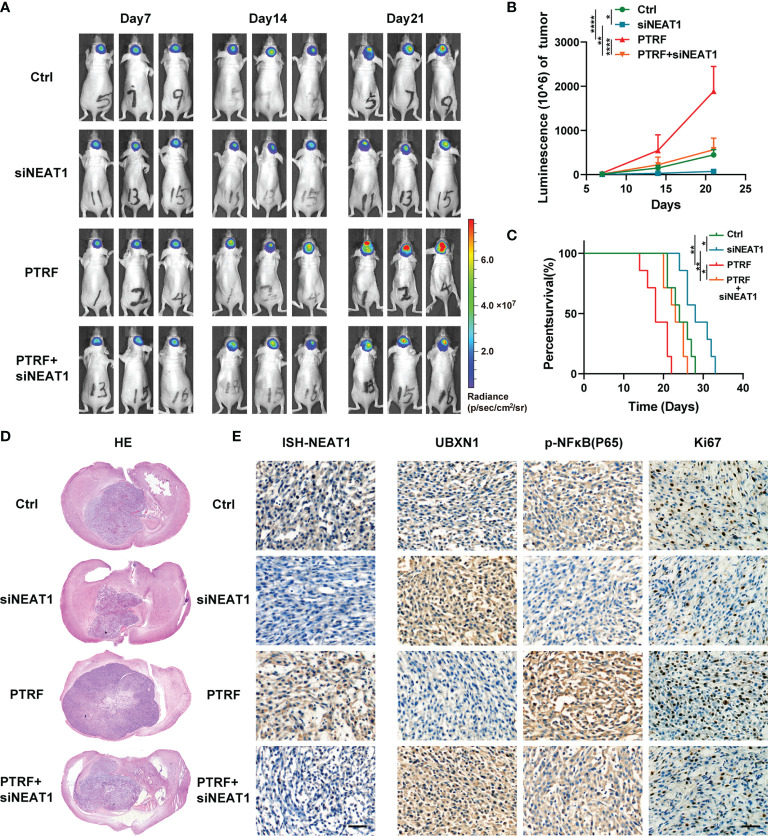
PTRF contributes to GBM proliferation through the UBXN1/NF-κB axis by regulating *NEAT1 In Vivo*. **(A)** Representative tumor bioluminescence images of mice at 7, 14, and 21 days post tumor implantation. **(B)** Tumor growth curves using quantification of bioluminescence imaging signal intensities for mice bearing GBM with or without PTRF overexpression, as well as treatment with *NEAT1* knockdown respectively. **(C)** Kaplan-Meier survival curve of nude mice implanted with TBD0220 cells with or without PTRF overexpression, as well as treatment with *NEAT1* knockdown respectively. **(D)** Representative images of H&E staining of tumors from the nude mice. **(E)** ISH staining of *NEAT1* in tumors (left). IHC staining of UBXN1, P-NF-κB, and Ki-67 in tumors (right). Scale bar, 50 μm. *p ≤ 0.05, **p ≤ 0.01, and ****p ≤ 0.001.

To further verify the role of the PTRF/*NEAT1*/NF-κB axis *in vivo*, we established an intracranial GBM patient-derived xenografts model in BALB/c nude mice. We transfected TBD0220 and TBD0220 PTRF cells with *NEAT1* siRNA respectively. The bioluminescence imaging showed that PTRF overexpression resulted in a significant increase in tumor growth. *NEAT1* knockdown reduced the growth of the tumor and attenuated the tumor growth induced by PTRF (**Figures 4A, B**). The mice bearing tumors, derived from the PTRF overexpressing cells, showed poor survival compared with the control group by Kaplan–Meier survival curves. *NEAT1* knockdown group had a longer survival time compared with their respective control groups (**Figure 4C**). Similar results could be found in Hematoxylin-Eosin (H&E) staining that *NEAT1* knockdown partly reverse the promotion of GBM proliferation induced by PTRF (**Figure 4D**). The expression of *NEAT1* was increased in the PTRF overexpressing group compared with the control group (**Figure 4E**). Overexpression of PTRF resulted in a significant decrease in UBXN1 and an increase in Ki-67 and p-NF-κB (**Figure 4E**). *NEAT1* knockdown significantly increased the level of UBXN1 and decreased the level of Ki-67 and p-NF-κB (**Figure 4E**). These data suggest that PTRF regulates *NEAT1* and promotes GBM proliferation through the UBXN1/NF-κB axis.

### The NF-κB Binds to the PD-L1 Promoter Region in Response to PTRF Activation and Enhances PD-L1 Transcription

**Figure 5:**
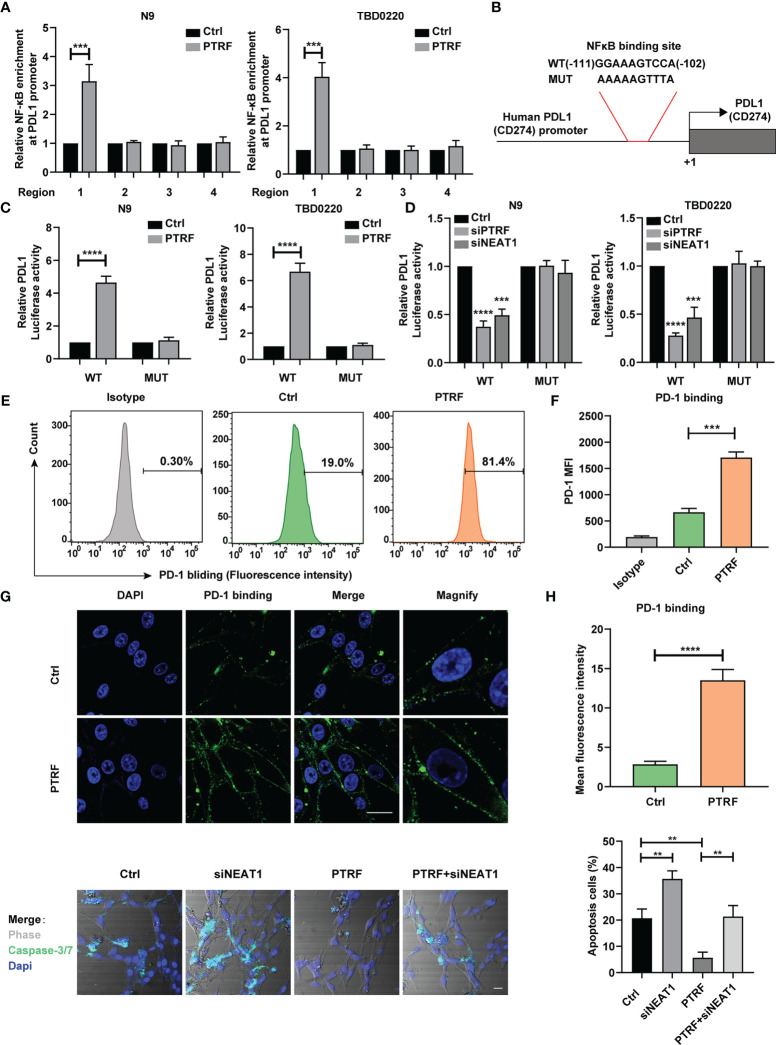
PTRF enhances PD-L1 transcription *via* NF-κB and promotes immune evasion of GBM cells through PD-L1. **(A)** ChIP-PCR analysis of the ability of NF-κB to bind to the PD-L1 promoter in N9 and TBD0220 cells with or without PTRF overexpression. Results are normalized to IgG. **(B)** Schematic illustration of the proximal region of the human PD-L1 promoter. **(C)** N9 and TBD0220 cells (with the expression of empty vector or PTRF) were transfected with a luciferase reporter vector containing WT or mutated NF-κB sequence of PD-L1 promoter. **(D)** N9 and TBD0220 cells with PTRF or *NEAT1* knockdown were transfected with a luciferase reporter vector containing WT or mutated NF-κB sequence of PD-L1 promoter. **(E)** Flow cytometry analysis of PD-1 binding in TBD0220 cells with or without PTRF overexpression. **(F)** Quantification of fluorescence intensity in **(E)**. The y axis represents the mean fluorescence intensity of PD-1. **(G)** Immunofluorescence staining of control and PTRF overexpressing cells. PD-1 (green), Nuclei (blue), scale bar, 20 μm. **(H)** Quantification of fluorescence intensity in **(G)**. **(I)** T cell killing assay of TBD0220 cells with or without PTRF overexpression, as well as treatment with *NEAT1* knockdown respectively. caspase3/7 cleavage (green), Nuclei (blue), scale bar, 20 μm. **p ≤ 0.01, ***p ≤ 0.001, and ****p ≤ 0.001.

To identify the binding sequence of NF-κB in the promoter region of PD-L1, we analyzed the PD-L1 promoter and found four putative binding sites for NF-κB in the PD-L1 promoter region (25). Four pairs of primers with CHIP-qPCR for PD-L1 promoter were designed respectively. The sonicated chromatin from PTRF overexpression in N9 and TBD0220 cells was immunoprecipitated with an anti-NF-κB antibody, and only one fragment was amplified in N9 PTRF and TBD0220 PTRF cells compared with control cells by PCR, indicating (–111)-GGAAAGTCCA- (–102) as a consensus sequence recognized by NF-κB (**Figure 5A**). We constructed a luciferase reporter driven by the PD-L1 promoter that contains the WT or a mutated potential NF-κB-binding sequence (**Figure 5B**). We transfected the luciferase reporter construct into N9 and TBD0220 cells and measured luciferase activity. PTRF overexpression in N9 and TBD0220 cells significantly increased the activity of the WT but not of mutated promoter, indicating that PTRF increased the binding of NF-κB to PD-L1. The knockdown of PTRF reduced the luciferase activity driven by the PD-L1 promoter in N9 and TBD0220 cells (**Figure 5C**). A similar inhibitory effect was also observed by *NEAT1* knockdown in N9 and TBD0220 cells (**Figure 5D**). These results demonstrate that NF-κB binds to the PD-L1 promoter region and enhances the PD-L1 transcription in response to PTRF activation in GBM cells, and this regulation is mediated *via* one of the NF-kB binding sites (-111 to -102 nt region).

### PTRF Promotes Immune Evasion of GBM Cells by Affecting PD-1 Binding and PD-L1–Mediated T Cell Toxicity

Previous studies have shown that elevated PD-L1 expression in tumor cells mediates T cell tolerance and activates anti-tumor immunosuppression (10, 12). To determine whether PTRF regulation on PD-L1 may affect its interaction with PD-1, we incubated a recombinant protein containing human PD-1 and Fc fragment of IgG with TBD0220 cells. We found that PTRF overexpression increased PD-1 binding on the TBD0220 cell surface compared with the control cells (**Figures 5E, F**). Immunofluorescence assays revealed that overexpression of PTRF was accompanied by high expression of PD-1 (**Figures 5G, H**), indicating that PTRF significantly increased PD-1 binding on the GBM cell surface, which is consistent with the flow cytometry results. Furthermore, PTRF overexpression decreased the sensitivity of GBM cells to T cell-mediated cytotoxicity. The knockdown of *NEAT1* sensitized GBM cells to T cell-mediated cell death and attenuated the immune evasion induced by PTRF in GBM (**Figure 5I**). These results demonstrate that PTRF alters PD-1 binding and T cell-mediated cytotoxicity through regulation of PD-L1 expression in GBM.

**Figure 6:**
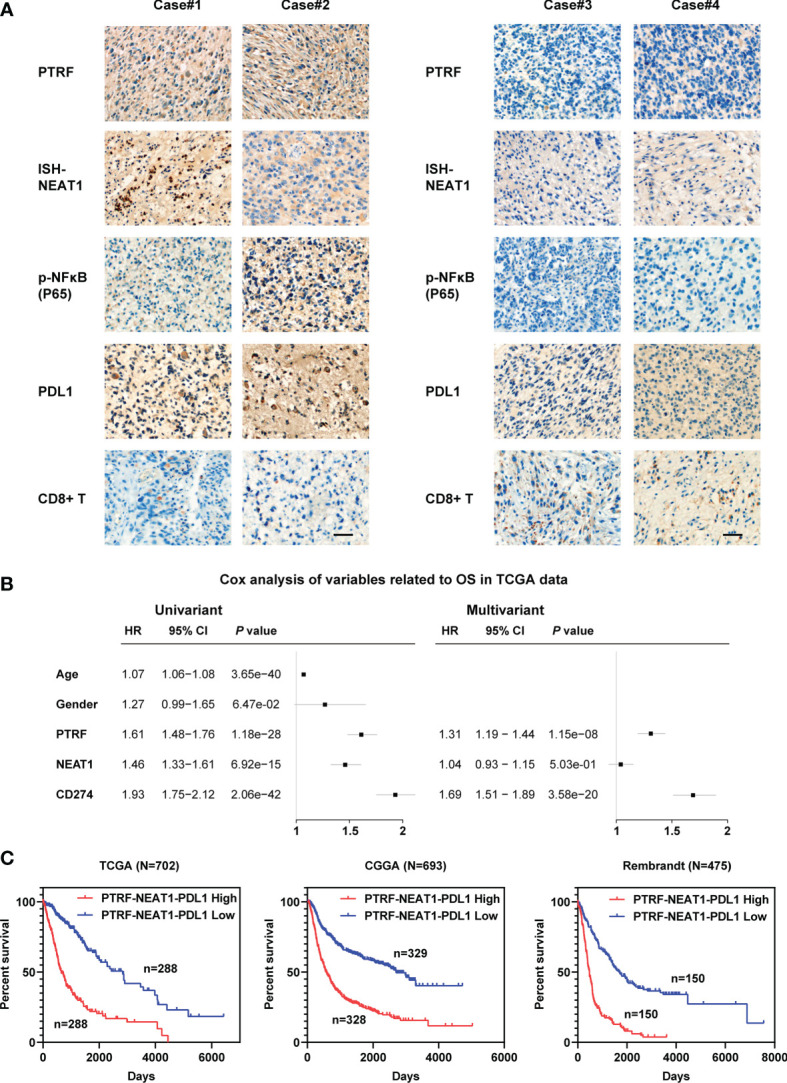
The relationships between PTRF, *NEAT1*, and PD-L1 in GBM. **(A)** IHC or ISH staining of PTRF, *NEAT1*, P-NF-Kb, PD-L1, and CD8 in four representative GBM specimens. Scale bar, 50 μm. **(B)** Univariate and multivariate Cox analyses of PTRF, *NEAT1*, and PD-L1 expression in the TCGA databases. **(C)** The Kaplan–Meier curves for patients with different expressions of PTRF, *NEAT1*, and PD-L1.

To further assess relationships between PTRF, *NEAT1*, and PD-L1 in GBM, we chose tissue samples of GBM patients with high or low PTRF expression. GBM patients with high PTRF-expression had high levels of *NEAT1* and p-NF-κB, showing that PTRF significantly correlates with *NEAT1* and p-NF-κB (**Figure 6A**). Furthermore, high levels of PTRF positively correlated with PD-L1 expression and inversely correlated with CD8^+^ T cell infiltration in GBM (**Figure 6A**). These results suggest a role of PTRF in PD-L1 regulation and immune evasion in GBM.

To evaluate the prognostic relevance of PTRF, *NEAT1*, and PD-L1 expression, we analyzed glioma data from TCGA, CGGA, and Rembrandt databases. As shown in **Figure 6B** and **Supplemental Figure 4**, high expressions of PTRF, *NEAT1*, and PD-L1 are independent poor prognostic markers. Next, we integrated the expression levels of these genes and evaluated the suggestive effect of the PNP geneset (PTRF-*NEAT1*-PD-L1) on the prognosis of GBM patients. We show that a higher level of PNP indicates a shorter survival time in patients with glioma in TCGA, CGGA, and Rembrandt databases (**Figure 6C**).

## Discussion

**Figure 7:**
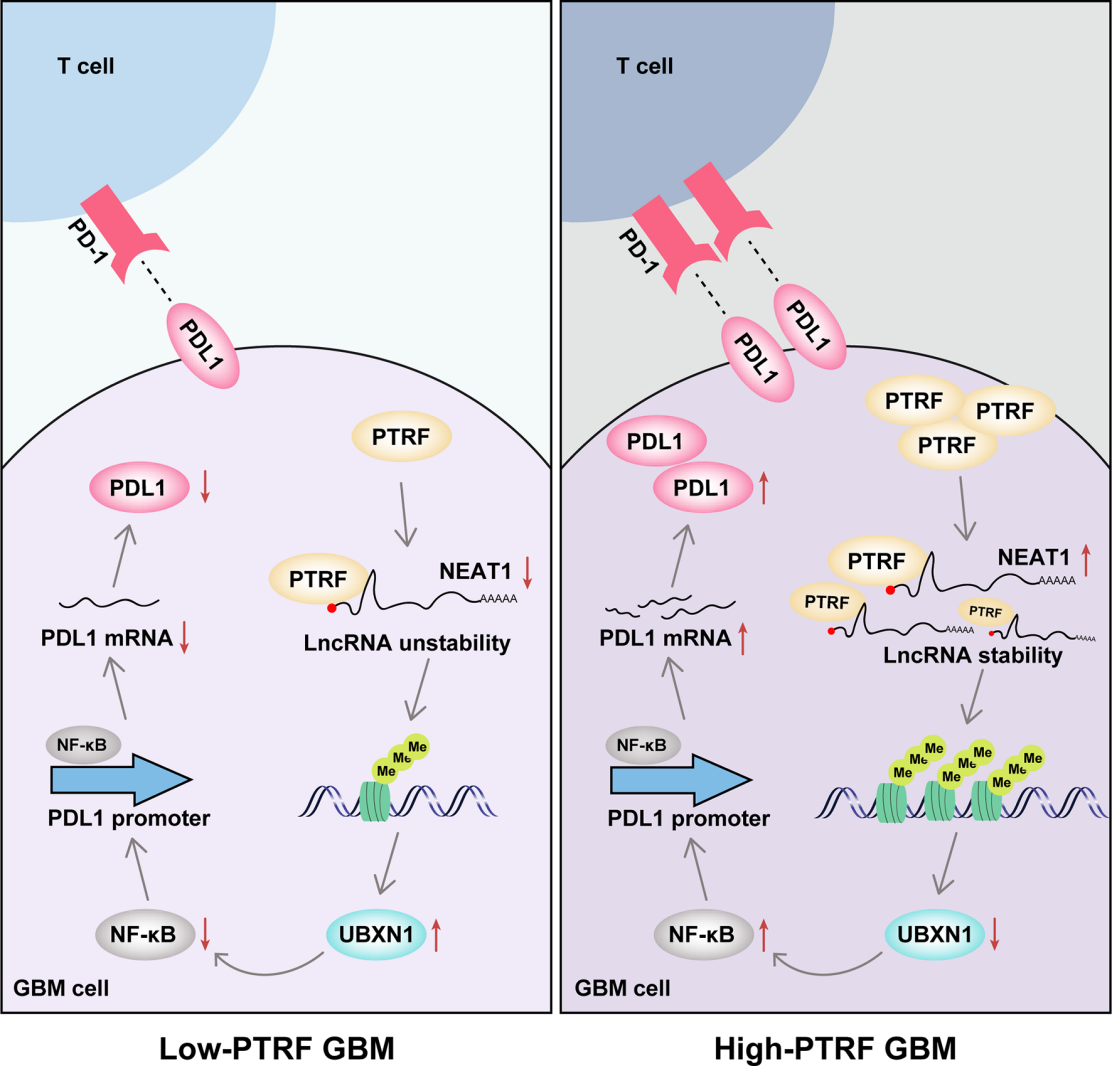
The mechanistic model showing how PTRF/Cavin-1 stabilizes lncRNA *NEAT1* to maintain the activity of NF-κB and promote immune evasion *via* PD-L1 in glioblastoma.

In this study, we show that PTRF induces the stability of lncRNA *NEAT1* and promotes the activity of NF-κB, leading to PD-L1 mediated immune evasion in GBM (**Figure 7**). According to previous studies, PTRF plays different roles in different types of tumors. PTRF inhibits tumor metastasis in prostate cancer and tumorigenesis in colorectal cancer (26, 27). In contrast, PTRF promotes the progression of pancreatic cancer and proliferation of rhabdomyosarcoma (28, 29). The tumor-promoting effect of PTRF in gliomas is widely recognized. Previously, we showed that the expression of PTRF positively correlates with the WHO grade of glioma, and the prognosis of glioma patients with high PTRF expression is worse (15). We further found a reduction in the number of CD8^+^ T cells and CD8^+^ T cells mediated cytotoxic activity in tumor tissues with PTRF overexpression (19). In this study, we demonstrated that PTRF increases the expression of PD-L1 and promotes immune evasion in GBM.

Recently studies have shown that lncRNAs play a crucial roles in regulating immunity, including the activation and function of macrophages and T cells (30). One such lncRNA is *NEAT1*. Previous studies have found that *NEAT1*-knockout mice have abnormalities in their Treg and Th cell differentiation (31). *NEAT1* has also been found to target STAT3 and regulate the differentiation of Th17 cells in rheumatoid arthritis (32). Imamura et al. found that *NEAT1* activate the transcription of IL-8 by removing SFPQ from the IL-8 promoter (33). Knockdown of *NEAT1* also limits the apoptosis of CD8^+^ T cells through the miR-155/Tim-3 pathway (34). Although *NEAT1* has been studied in immune response, the relationship between *NEAT1* and PD-L1 remains unclear. In this study, we found that *NEAT1*, induced by PTRF, mediates PD-L1 regulation and promotes immune evasion in GBM.

UBXN1 is a UBX domain-containing protein which inhibits NF-κB activation. The knockdown of UBXN1 decreases the stability of IκBα and enhances NF-κB signaling (35). In addition, UBXN1 is the negative regulator of NF-κB signaling and interacts with cIAPs through the UBA domain (36). YTHDF2 was also shown to accelerate UBXN1 mRNA degradation *via* METTL3−mediated m6A, promoting the activation of NF−κB (37). Our previous study found that UBXN1 is also a target for EZH2 mediated H3K27me3, and is regulated by EGFR and lncRNA *PRADX* (23, 24). In addition, we also detected that lncRNA *NEAT1* binds to EZH2 and mediates the trimethylation of H3K27 in their promoters (38), which regulated by EGFR pathway activity. From the discussion above, we further demonstrated that PTRF maintains the stability of *NEAT1* to suppresses UBXN1 expression and activate NF-κB activity.

Although the expression of PD-L1 is regulated by multiple mechanisms, transcriptional regulation plays an important part in its regulation in cancer. MYC was shown to directly bind to the promoter of PD-L1 and enhanced the expression of PD-L1, regulating the anti-tumor response in mouse tumors and human tumor cells (39). Interferon regulatory factor-1 (IRF-1), a transcription factor, regulates the constitutive and IFN-γ-mediated PD-L1 expression in human lung cancer cell (40). In classical Hodgkin lymphoma, AP-1-responsive enhancer in the PD-L1 gene binds to the PD-L1 enhancer and increases the activity of PD-L1 promoter (41). Recently, MUC1-C was found to activate PD-L1 transcription by recruiting MYC and NF-κB to the PD-L1 promoter region in triple-negative breast cancer (42). Our study identified another novel mechanism of PD-L1 regulation in cancer—PTRF induces the binding of NF-κB to the PD-L1 gene promoter region and enhances PD-L1 transcription in GBM.

## Data Availability Statement

The data presented in the study are deposited in the SRA repository, accession number PRJNA777377.

## Ethics Statement

The animal study was reviewed and approved by the Animal Ethical and Welfare Committee (Protocol # TMUaMEC 2018034). Written informed consent was not obtained from the individual(s) for the publication of any potentially identifiable images or data included in this article.

## Author Contributions

Developed the study concept and designed the study: YT and QW. Conducted the experiments and acquired the data: KY, XC, XL, YW, JZ, SY, CX, EY, MX, BH, CF, YT and QW. Analyzed the data, generated the figures, and wrote the paper: CK, KY and XC. All authors contributed to the article and approved the submitted version.

## Funding

This work was supported by the National Natural Science Foundation of China (No. 81772667 and 82002657), the Tianjin Key R&D Plan of Tianjin Science and Technology Plan Project (No. 20YFZCSY00360), the Central Government Guided Local Science and Technology Development fund Project of Hebei Province (No. 216Z7711G), the Hebei Natural Science Foundation Precision Medicine Joint Project (No. H2020201206), the Key Scientific Research Project in Colleges and Universities of Hebei Province (No. ZD2021308), The National Key Research and Development Programs of China (No. 2018YFA0209700), and the Science and Technology Project of Tianjin Municipal Health Commission (No. TJWJ2021QN007 and TJWJ2021QN003).

## Conflict of Interest

The authors declare that the research was conducted in the absence of any commercial or financial relationships that could be construed as a potential conflict of interest.

## Publisher’s Note

All claims expressed in this article are solely those of the authors and do not necessarily represent those of their affiliated organizations, or those of the publisher, the editors and the reviewers. Any product that may be evaluated in this article, or claim that may be made by its manufacturer, is not guaranteed or endorsed by the publisher.
