# Supplementary material for: PTRF/Cavin-1 as a Novel RNA-Binding Protein Expedites the NF-κB/PD-L1 Axis by Stabilizing lncRNA NEAT1, Contributing to Tumorigenesis and Immune Evasion in Glioblastoma
Source: Front Immunol. 2022 Jan 6;12:802795. doi: 10.3389/fimmu.2021.802795 (PMC8778801; doi:10.3389/fimmu.2021.802795)
Supplement: Supplementary file 1 [file DataSheet_1.docx]

**Supplemental Material**

**
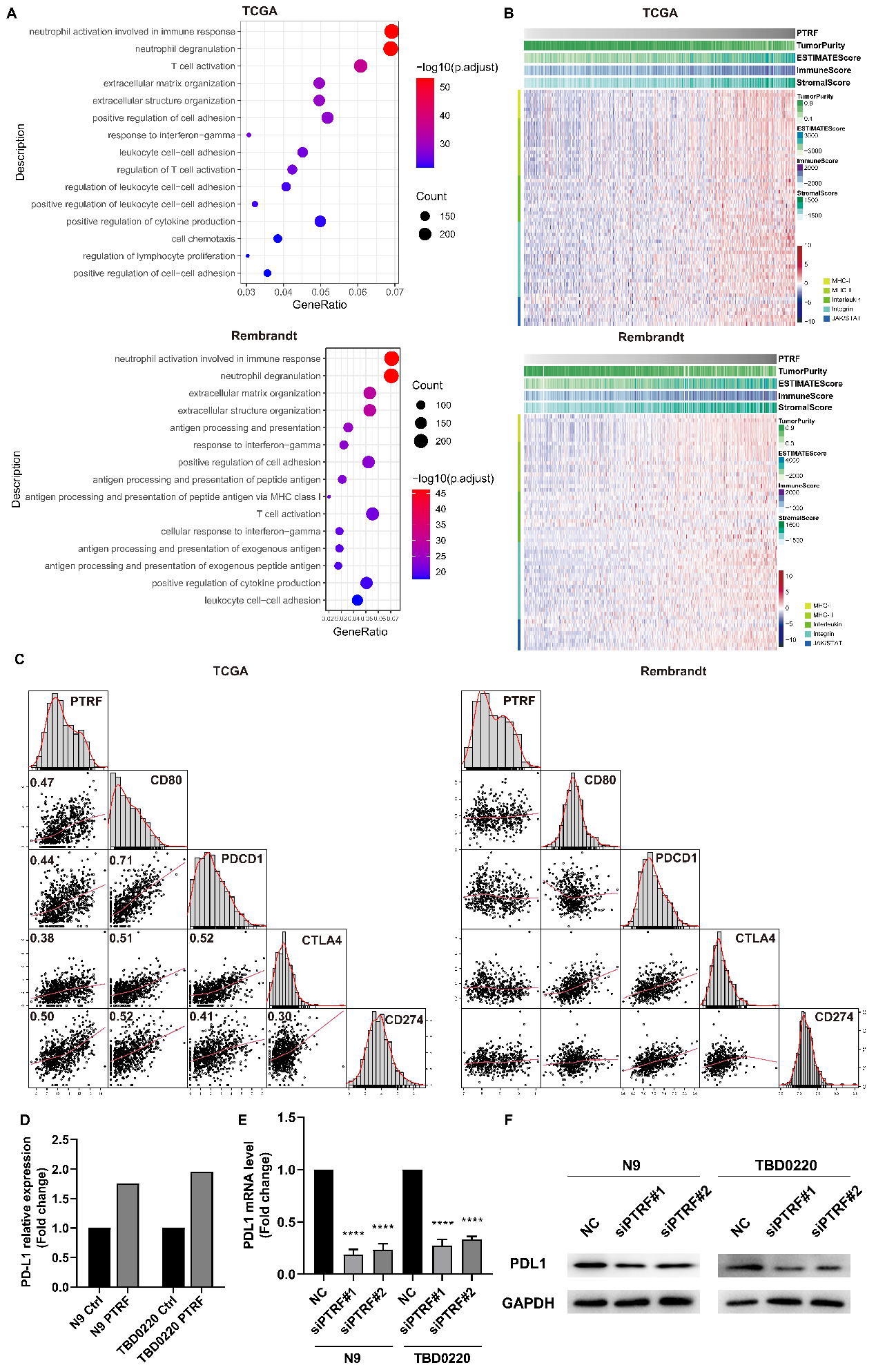
**

**Figure S1, related to Figure 1. PTRF increases PD-L1 levels and correlates with immunosuppression in GBM.** (A) Gene ontology enrichment analysis of genes that are positively associated with PTRF in the TGGA and Rembrandt database. (B) Heatmap showing the relationship between PTRF, low tumor purity, and genes involved in immunity in the TGGA and Rembrandt database. (C) The correlation between PTRF, CD80, PDCD1, CTLA4, and PD-L1 levels in the TGGA and Rembrandt database and measured by Pearson’s correlation test. (D) Relative level of PD-L1 in N9 and TBD0220 cells using ImageJ software. (E) Western blot analysis of PD-L1 expression in N9 and TBD0220 cells with PTRF knockdown. (F) Relative mRNA level of PD-L1 in N9 and TBD0220 cells with PTRF knockdown.


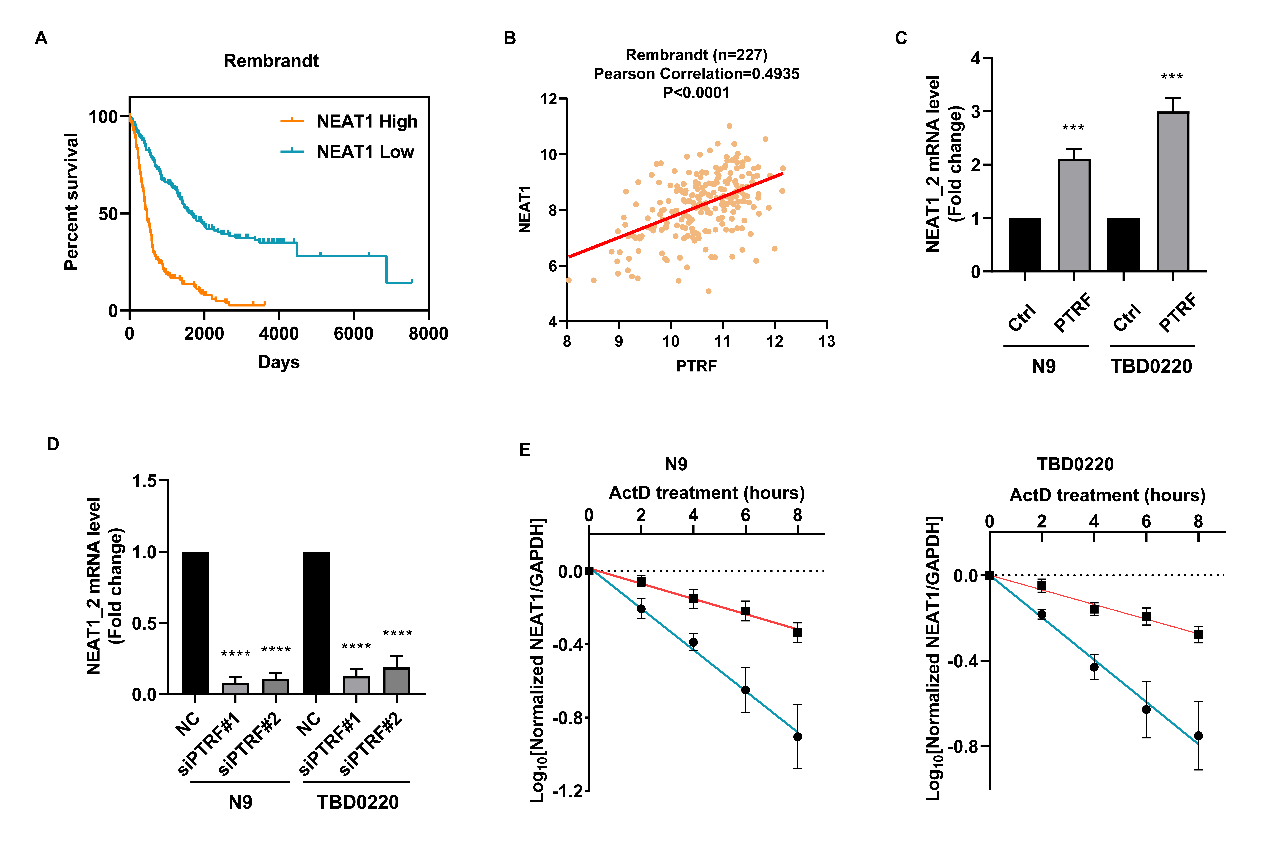


**Figure S2, related to Figure 2. PTRF interacts with LncRNA *NEAT1* and maintains its mRNA stability.** (A) The Kaplan–Meier curves of patients with high or low LncRNA *NEAT1* expression (Rembrandt database). (B) The correlation between PTRF and *NEAT1* levels in the Rembrandt database. (C) The relative level of *NEAT1* in control and PTRF overexpressing cells. (D) The level of *NEAT1* in N9 and TBD0220 cells with PTRF knockdown. (E) RT-qPCR analysis of *NEAT1* treated with Actinomycin D (ActD) at the indicated time points in N9 and TBD0220 cells.


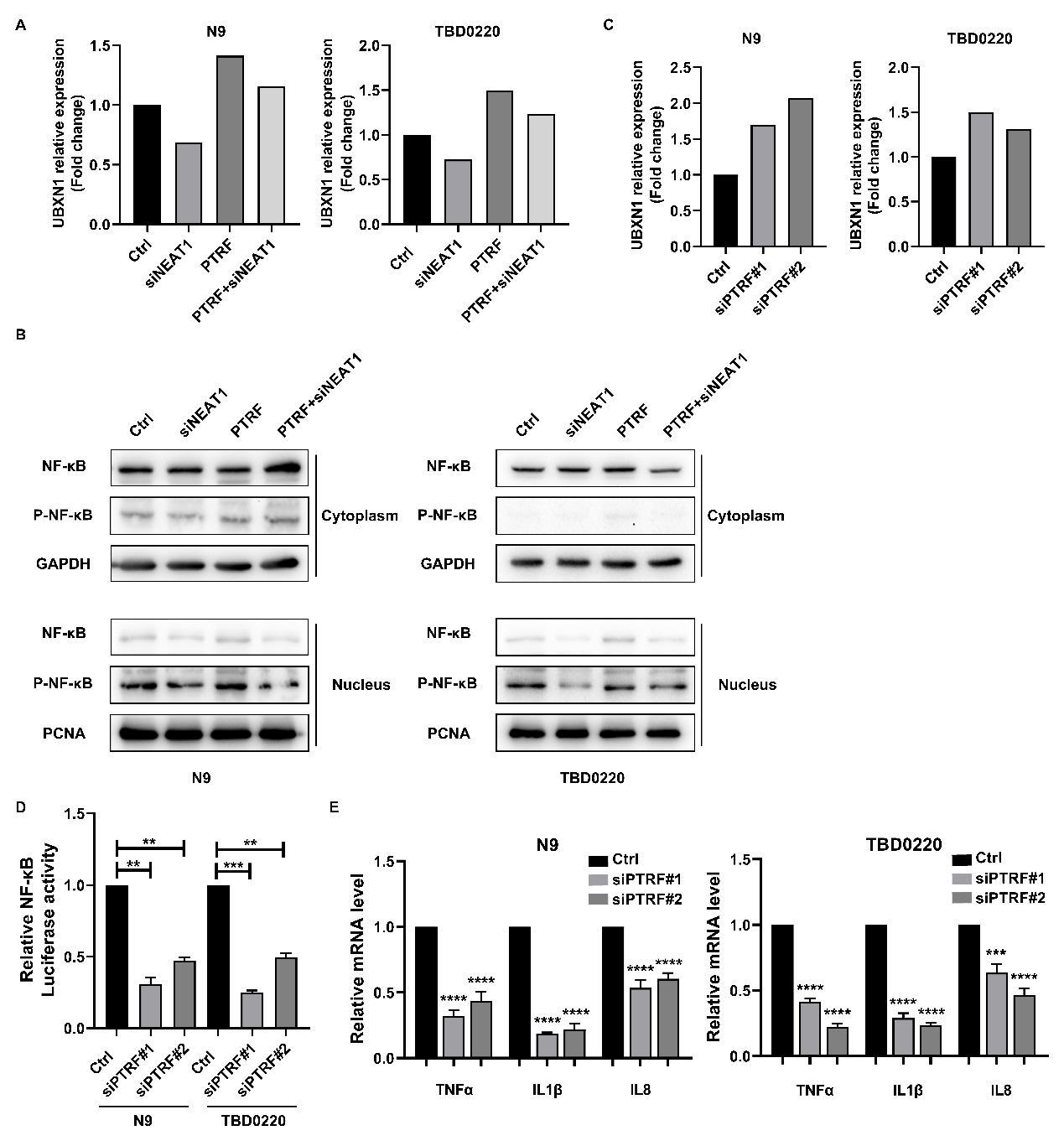


**Figure S3, related to Figure 3. PTRF suppresses UBXN1 expression and promotes the activity of NF-κB via *NEAT1*.** (A) Relative level of UBXN1 in Figure 3E. (B) Nuclear and cytoplasmic protein of NF-κB, and P-NF-κB in N9 and TBD0220 cells with or without PTRF overexpression, as well as treatment with NEAT1 knockdown respectively. (C) Relative level of UBXN1 in Figure 3F. (D) The Luciferase activity of NF-κB in N9 and TBD0220 cells with PTRF knockdown. (E) The mRNA level of NF-κB downstream target genes, including TNFα, IL-1β, and IL-8 in N9 and TBD0220 cells with PTRF knockdown.


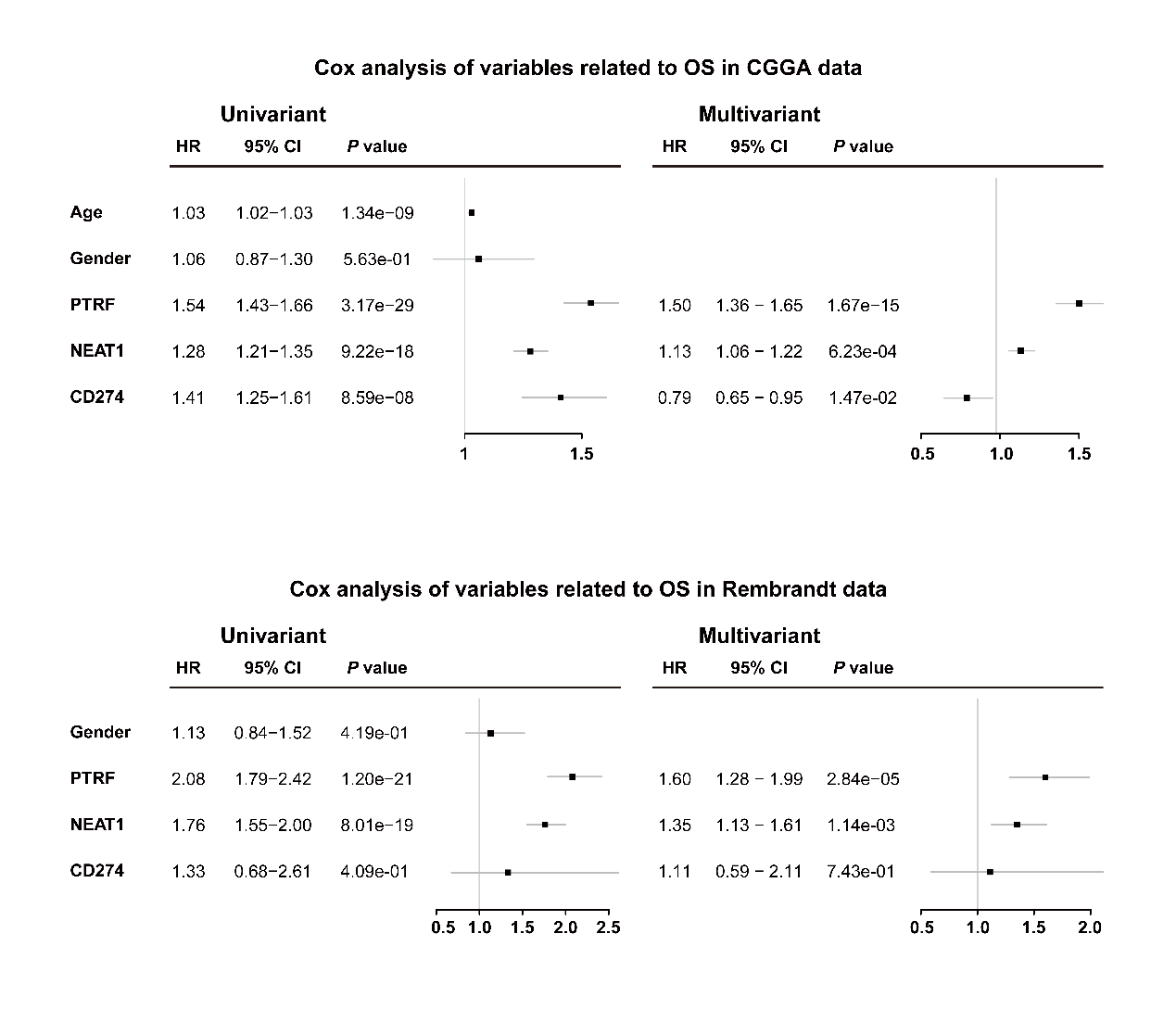


**Figure S4, related to Figure 6. The relationships between PTRF, *NEAT1*, and PD-L1 in GBM.** (A) Univariate and multivariate Cox analyses of PTRF, *NEAT1*, and PD-L1 expression in the CGGA and Rembrandt databases.
